# Association Between Relative Fat Mass and Cardiometabolic Disease: Age-Stratified Analysis in Young and Middle-Aged Versus Older Adults

**DOI:** 10.31083/RCM45938

**Published:** 2025-11-27

**Authors:** Teng Li, Xian Xie, Zening Jin, Jing Nan, Jing Han, Li Yin

**Affiliations:** ^1^Department of Cardiology and Macrovascular Disease, Beijing Tiantan Hospital, Capital Medical University, 100070 Beijing, China; ^2^Hunan Provincial Center for Disease Control and Prevention, 410153 Changsha, Hunan, China

**Keywords:** relative fat mass, cardiometabolic disease, young and middle-aged adults, older adults, dose-response relationship

## Abstract

**Background:**

Current evidence characterizing the association between relative fat mass (RFM) and cardiometabolic disease (CMD) remains limited, with critical gaps persisting in the understanding of age-dependent heterogeneity. Thus, this study aimed to assess the association between RFM and CMD risk across age groups.

**Methods:**

This study utilized data from the China Health Evaluation And Risk Reduction Through Nationwide Teamwork (ChinaHEART), and enrolled 93,801 community-dwelling adults. CMD was defined as a composite diagnosis that included diabetes mellitus, myocardial infarction, and stroke. Meanwhile, RFM was derived from height, waist circumference, and sex. Participants were stratified into groups of young and middle-aged adults (35–59 years) and older adults (≥60 years). Multivariable logistic regression models were employed to estimate odds ratios (ORs) and 95% confidence intervals (CIs), and to test for interaction effects. Restricted cubic spline models were applied to examine dose–response relationships.

**Results:**

Among the 93,801 participants, 18,473 (19.69%) had CMD. In the fully adjusted models, each unit increase in RFM was associated with a 9% increase in CMD risk (OR = 1.09, 95% CI: 1.08–1.09). Compared to the lowest RFM quartile (Q1), higher risks were observed in the Q2 (1.68, 1.59–1.77), Q3 (2.56, 2.34–2.80), and Q4 (4.02, 3.68–4.39) groups (*p* for trend <0.001). A significant RFM–age interaction was identified (*p* for interaction = 0.001). Restricted cubic splines confirmed significant non-linear dose–response relationships (both *p* for overall association <0.001; *p* for non-linear <0.05), with distinct age-specific patterns. Older adults exhibited higher overall CMD risk compared to young and middle-aged adults. The lower RFM inflection point corresponds to an OR of 1 (30 vs. 34), highlighting the greater vulnerability of this age group and informing the future development of age-specific RFM thresholds.

**Conclusions:**

RFM demonstrates a significant positive association with CMD risk, exhibiting age-dependent heterogeneity, and emphasizing age-tailored interventions for CMD prevention strategies.

## 1. Introduction

Cardiometabolic diseases (CMD), including diabetes, myocardial infarction, and stroke, pose a growing public health threat due to their increasing prevalence [1, 2, 3]. These conditions share pathophysiologies such as metabolic inflammation and ectopic lipid deposition, and often presenting with overlapping therapeutic targets [4, 5, 6]. With CMD prevalence rising with age [7, 8] and against the backdrop of global population aging, there is a pressing need for simple, accurate indicators to predict the risk of CMD and guide interventions. Traditional anthropometric measures like body mass index (BMI) and waist circumference, while associated with CMD risk, fail to distinguish fat from lean mass [9]. Advanced techniques such as dual-energy X-ray absorptiometry (DXA) and bioelectrical impedance analysis (BIA) have limitations in clinical practice due to cost and complexity.

Relative fat mass (RFM), a novel anthropometric metric derived from height, waist circumference, and sex, serves as a validated indicator of adiposity with strong correlations to DXA- and BIA-measured body fat percentage [9, 10]. Prior studies have linked RFM to increased risks of coronary heart disease [11], stroke [12], type 2 diabetes [13, 14, 15], metabolic syndrome [16], and heart failure [17]. RFM may be superior to BMI in predicting the risk of diabetes [13, 14, 15] and the metabolic syndrome [16]. However, evidence on the association between RFM and CMD remains scarce, particularly regarding dose-response relationships and age-specific variations. Aging-driven mechanisms—including ectopic fat redistribution [18], chronic inflammation [19], and multifaceted insulin resistance [20]—suggest potential heterogeneity in the association between RFM and CMD across age groups, however, stratified analyses are currently lacking.

We analyzed the data from China Health Evaluation And Risk Reduction Through Nationwide Teamwork (ChinaHEART), a large scale, population-based study covering all 31 provinces in mainland China, to investigate the relationship between RFM and CMD and evaluate its variation across age groups (young and middle-aged vs. older adults).

## 2. Materials and Methods

### 2.1 Study Design and Population

**Fig. 1.**
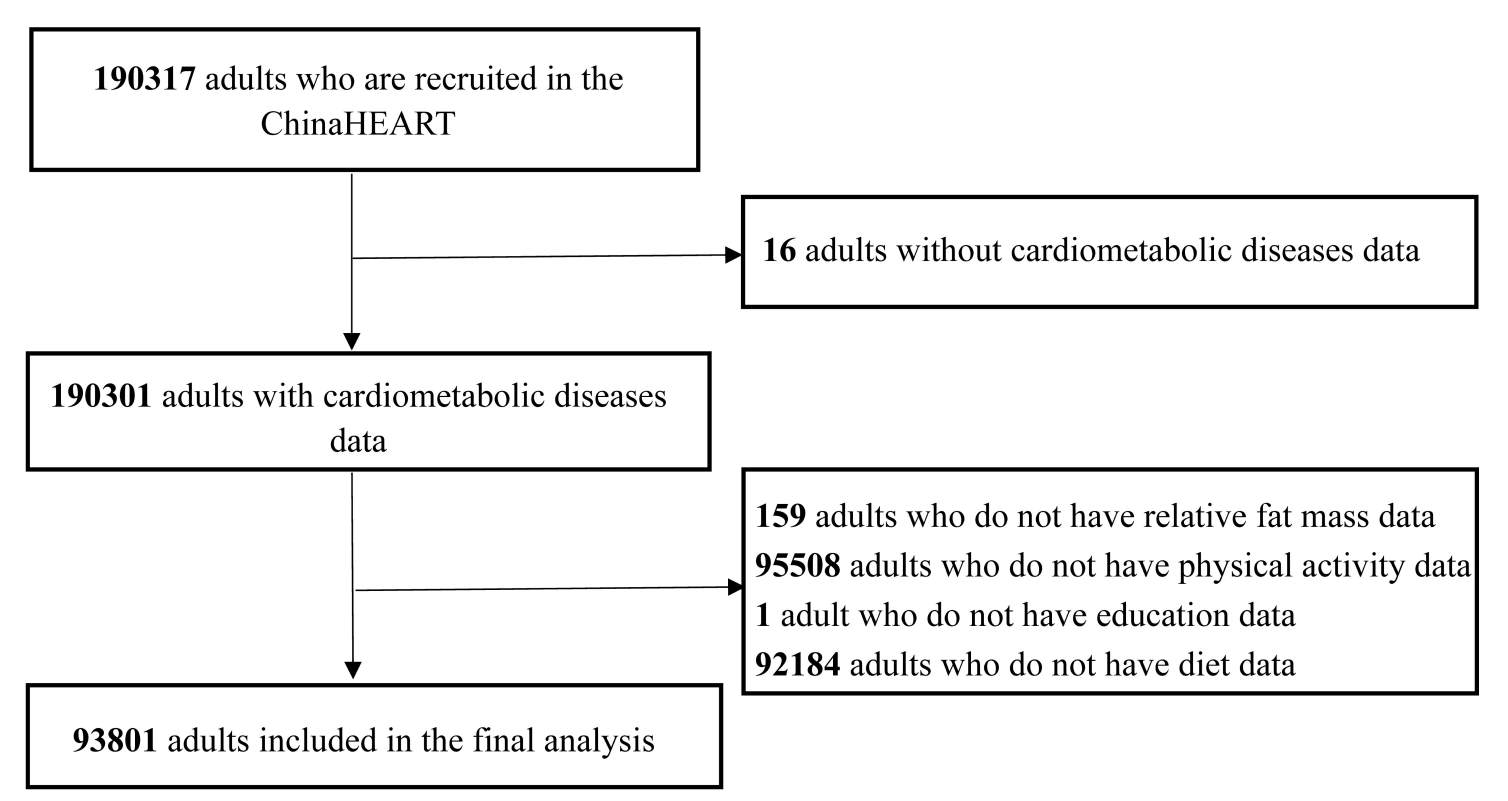
**Study enrollment flowchart**.

The ChinaHEART, a nationwide public health project, served as the data source. Detailed protocols have been published previously [21]. From 2016 to 2023, we enrolled 190,317 community-dwelling adults aged 35–75 years across 20 sites in Hunan Province, China. Participants completed standardized questionnaires (demographics, lifestyle, medical history, *ect*.) and underwent physical/laboratory examinations. After excluding individuals with missing key variables (anthropometrics, CMD status, socioeconomic factors, lifestyle characteristics, *ect*.), 93,801 participants were retained for analysis (Fig. 1).

Ethical approval was granted by Fuwai Hospital’s Institutional Review Board (No. 2014-574). All participants provided written informed consent.

### 2.2 Data Collection and Definition 

Trained staff collected data using standardized protocols: (1) Questionnaires: Demographics, socioeconomic status (annual household income: ≥¥10,000 (US $1,408) vs. <¥10,000; education: middle school and above vs. primary school and below), lifestyle (smoking: current/never; alcohol: frequent [more than 4 times per week] vs. non-frequent [never, once or less per month, 2–4 times per month, 2–3 times per week]) [22, 23], physical activity (meeting WHO guidelines [24]: yes/no), and diet (healthy/unhealthy per Chinese dietary guidelines [25]). (2) Anthropometrics: Height and waist circumference (cm) were measured using calibrated stadiometers with participants wearing lightweight clothing and having removed footwear and headwear [21]. RFM was calculated as: RFM = 64 – [20 × height (m) ÷ waist circumference (m)] + (12 × sex), where sex = 1 (female) or 0 (male) [10]. Height and waist circumference were measured in centimeters but converted to meters for the RFM calculation. (3) Laboratory tests and medical history: Hypertension was defined as systolic/diastolic BP ≥140/90 mmHg, self-reported diagnosis, or antihypertensive use. Dyslipidemia [26] required TC ≥6.2 mmol/L, LDL-C ≥4.1 mmol/L, HDL-C <1.0 mmol/L, TG ≥2.3 mmol/L, or lipid-lowering medication.

### 2.3 CMD Defination

CMD was defined as ≥1 of the following [4, 5, 6]: (1) Self-reported diabetes, or with the use of hypoglycemic agents/insulin; (2) Self-reported myocardial infarction; (3) Self-reported stroke.

### 2.4 Statistical Analysis 

Baseline characteristics were described for the total study population, young and middle-aged group (35–59 years), and older adult group (≥60 years), including socioeconomic characteristics, lifestyle information, and medical history. Continuous variables (age, RFM) were tested for normality using the Kolmogorov-Smirnov test, which indicated non-normal distributions; therefore, these variables were presented as median (interquartile range) and compared between groups using the Wilcoxon rank-sum test. Categorical variables (sex, annual household income, education level, smoking status, alcohol consumption, physical activity level, diet, hypertension, and dyslipidemia) were presented as frequency (percentage) and compared using chi-square tests.

Multivariable logistic regression models were used to calculate odds ratios (ORs) and 95% confidence intervals (CIs) for the association between RFM and CMD risk. RFM was analyzed as a continuous variable to calculate ORs and 95% CIs. Then, participants were divided into quartiles (Q1–Q4) based on RFM values, with Q1 as the reference group, to calculate ORs and 95% CIs for Q2, Q3, and Q4 groups. The logistic regression models were adjusted as follows: Model 1 adjusted for age and sex; the full model additionally adjusted for annual household income, education level, smoking status, alcohol consumption, physical activity level, diet, hypertension, and dyslipidemia.

The interaction between RFM and age group (<60 vs. ≥60 years) was tested in the full model. Stratified analyses were then performed in young and middle-aged, and older adult groups separately to examine the association between RFM and CMD risk, with results presented in forest plots for comparison. Restricted cubic spline functions were used to analyze dose-response relationships between RFM and CMD risk in each age group, adjusting for age, sex, annual household income, education level, smoking status, alcohol consumption, level of physical activity, diet, hypertension, and dyslipidemia.

All statistical analyses were performed using SAS 9.4 (SAS Institute Inc., Cary, NC, USA) and R 4.4.3 software (R Foundation for Statistical Computing, Vienna, Austria). Two-tailed tests were used, with *p* < 0.05 considered statistically significant.

## 3. Results

### 3.1 Baseline Characteristics

This study enrolled a total of 93,801 participants (Table 1), with a median age of 59 years (interquartile range [IQR]: 52, 67). The cohort comprised 47,004 (50.11%) middle-aged and young participants and 46,797 (48.89%) older participants. No significant difference was observed in RFM distribution between the two age groups (*p* = 0.739), with median [IQR] values of 34.44 [27.26, 38.75] and 34.06 [26.12, 39.78], respectively.

**Table 1.** **Baseline characteristics of the study population stratified by age groups**.

		Overall	≥60 years	<60 years	*p* value
Number of participants		93,801	46,797 (48.89%)	47,004 (50.11%)	
Age (years)		59.00 [52.00, 67.00]	67.00 [63.00, 70.00]	52.00 [47.00, 55.00]	*p * < 0.001
RFM		34.33 [26.67, 39.25]	34.06 [26.12, 39.78]	34.44 [27.26, 38.75]	*p* = 0.739
Sociodemographic characteristics					
	Gender (female)	56,601 (60.34%)	26,433 (56.48%)	30,168 (64.18%)	*p * < 0.001
Income					*p * < 0.001
	<¥10,000/year	9685 (10.33%)	7272 (15.54%)	2413 (5.13%)	
	≥¥10,000/year	81,391 (86.77%)	38,054 (81.32%)	43,337 (92.20%)	
	Unknown	2725 (2.91%)	1471 (3.14%)	1254 (2.67%)	
Education					*p * < 0.001
	Middle school and above	41,338 (44.07%)	12,965 (27.70%)	28,373 (60.36%)	
	Primary school and below	52,410 (55.87%)	33,804 (72.24%)	18,606 (39.58%)	
	Unknown	53 (0.06%)	28 (0.06%)	25 (0.05%)	
Lifestyle characteristics					
	Insufficient physical activity	72,001 (76.76%)	35,621 (76.12%)	36,380 (77.40%)	*p * < 0.001
	Unhealthy diet	87,701 (93.50%)	43,944 (93.90%)	43,757 (93.09%)	*p * < 0.001
	Alcohol consumption	6730 (7.17%)	4311 (9.21%)	2419 (5.15%)	*p * < 0.001
	Current smoking	22,188 (23.65%)	11,472 (24.51%)	10,716 (22.80%)	*p * < 0.001
Metabolic risk factors					
	Hypertension	53,283 (56.80%)	31,696 (67.73 %)	21,587 (45.93%)	*p * < 0.001
	Dyslipidemia	17,381 (18.53%)	9253 (19.77%)	8128 (17.29%)	*p * < 0.001

Age and Relative Fat Mass (RFM) are presented as median (interquartile range, IQR) due to non-normal distributions. Categorical variables are expressed as n (%). “Unknown” indicate participants who “declined to respond” or “were unaware of the answer”. ¥10,000 ≈ US $1408.

Compared with the older group, the middle-aged and young group demonstrated significantly higher proportions of female participants (64.18% vs. 56.48%), annual household income ≥¥10,000 (92.20% vs. 81.32%), educational attainment of middle school and above (60.36% vs. 27.70%), and insufficient physical activity (77.40% vs. 76.12%) (all *p* < 0.001). The older group exhibited a significantly higher prevalence of alcohol consumption (9.21% vs. 5.15%), and current smoking (24.51% vs. 22.80%) compared to the middle-aged and young group (all *p* < 0.001).

### 3.2 Association Between RFM and CMD Risk

In the total population, RFM demonstrated significant associations with increased CMD risk (Table 2). In the unadjusted model, a 1-unit increase in RFM was associated with a 3% higher risk of CMD (*p* < 0.001). After adjusting for age and sex, the OR was 1.10 (95% CI: 1.10–1.11, *p <*0.001), which remained significant in the multivariable-adjusted model (1.09, 1.08–1.09, *p* < 0.001). When RFM was categorized by quartiles, compared with the Q1 group, the Q2 (1.68, 1.59–1.77, *p* < 0.001), Q3 (2.56, 2.34–2.80, *p* < 0.001), and Q4 groups (4.02, 3.68–4.39, *p* < 0.001) all exhibited significantly higher risk of CMD (*p* for trend <0.001).

**Table 2.** **Logistic regression analysis of the association between RFM and CMD risk**.

		Unadjusted model		Adjusted for age and gender		Multivariable–adjusted	
		OR (95% CI)	*p*	OR (95% CI)	*p*	OR (95% CI)	*p*
RFM		1.03 (1.02–1.03)	<0.001	1.10 (1.10–1.11)	<0.001	1.09 (1.08–1.09)	<0.001
RFM Group							
	Q1	1.00		1.00		1.00	
	Q2	1.29 (1.24–1.36)	<0.001	1.90 (1.80–1.99)	<0.001	1.68 (1.59–1.77)	<0.001
	Q3	0.98 (0.94–1.03)	0.518	3.06 (2.80–3.34)	<0.001	2.56 (2.34–2.80)	<0.001
	Q4	1.80 (1.72–1.89)	<0.001	5.15 (4.72–5.62)	<0.001	4.02 (3.68–4.39)	<0.001
*p* for trend			<0.001		<0.001		<0.001

Abbreviations: CI, confidence interval; CMD, cardiometabolic disease; OR, odds ratio; RFM, relative fat mass; Q1–Q4, quartile groups stratified by RFM interquartile ranges. The multivariable-adjusted model included age, sex, annual household income, educational attainment, alcohol consumption, smoking status, physical activity, diet, hypertension, and dyslipidemia.

**Fig. 2.**
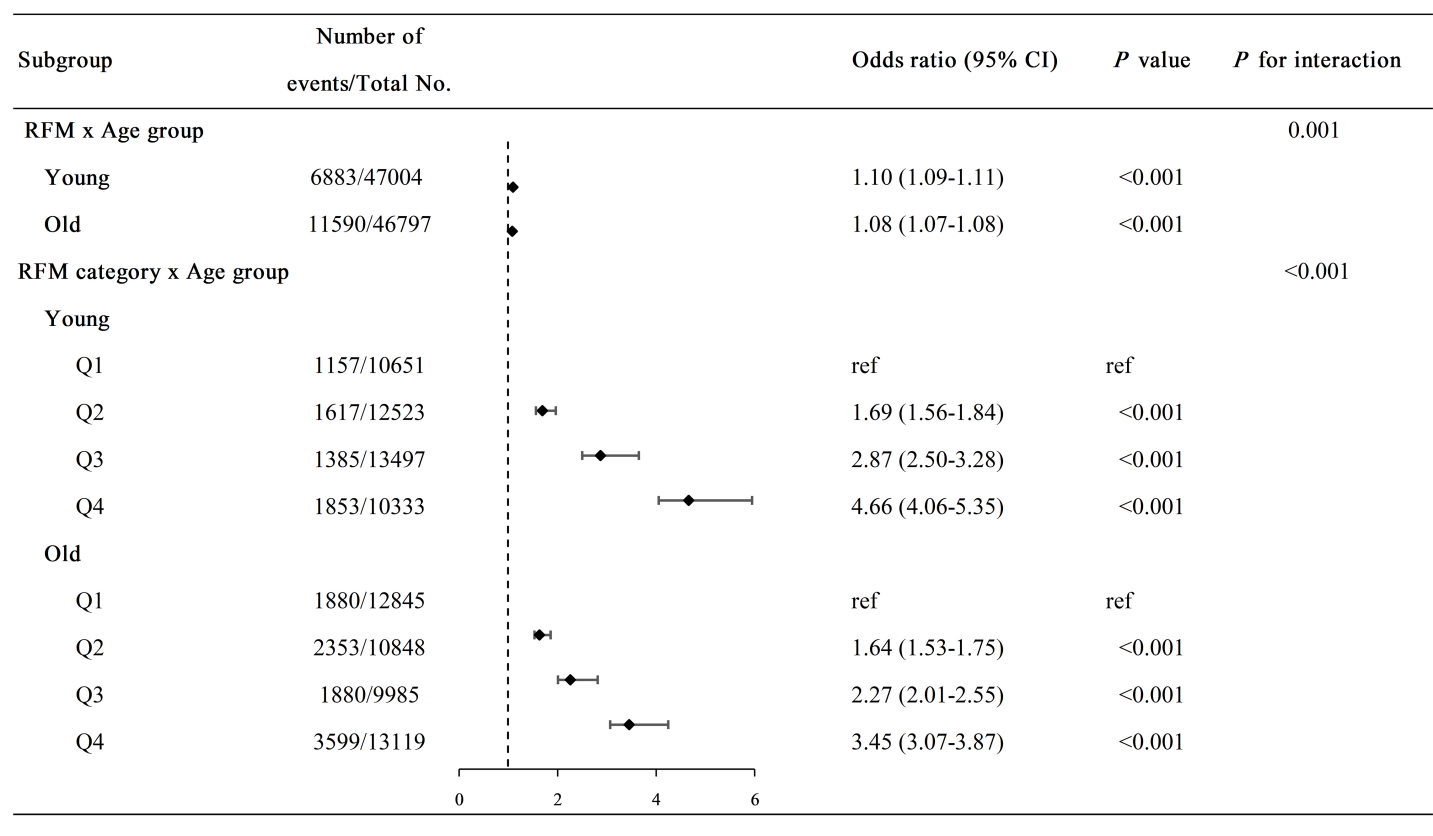
**Forest plot of subgroup analysis for the association between relative fat mass and cardiometabolic disease risk**. Young: age <60 years group; Old: age ≥60 years group. Abbreviations: CI, confidence interval; CMD, cardiometabolic disease; OR, odds ratio; RFM, relative fat mass; Q1–Q4, quartile groups stratified by RFM interquartile ranges. Model adjusted for age, sex, annual household income, educational attainment, alcohol consumption, smoking status, physical activity, diet, hypertension, and dyslipidemia.

Significant interaction effects were observed between RFM (*p* = 0.001) and RFM quartiles (*p* < 0.001) with age groups in the multivariable logistic regression model (Fig. 2). A 1-unit increase in RFM was associated with CMD risk elevation in the middle-aged and young group (1.10, 1.09–1.11, *p* < 0.001), as well as the older group (1.08, 1.07–1.08, *p* < 0.001). When stratified by RFM quartiles, both age groups showed progressively increased CMD risks across higher quartiles (vs. Q1): Q2 (young and middle-aged: 1.69, 1.56–1.84 vs. older: 1.64, 1.53–1.75), Q3 (2.87, 2.50–3.28 vs. 2.27, 2.01–2.55), and Q4 (4.66, 4.06–5.35 vs. 3.45, 3.07–3.87) (all *p* < 0.001).

### 3.3 Dose-Response Relationship Between RFM and CMD Risk

**Fig. 3.**
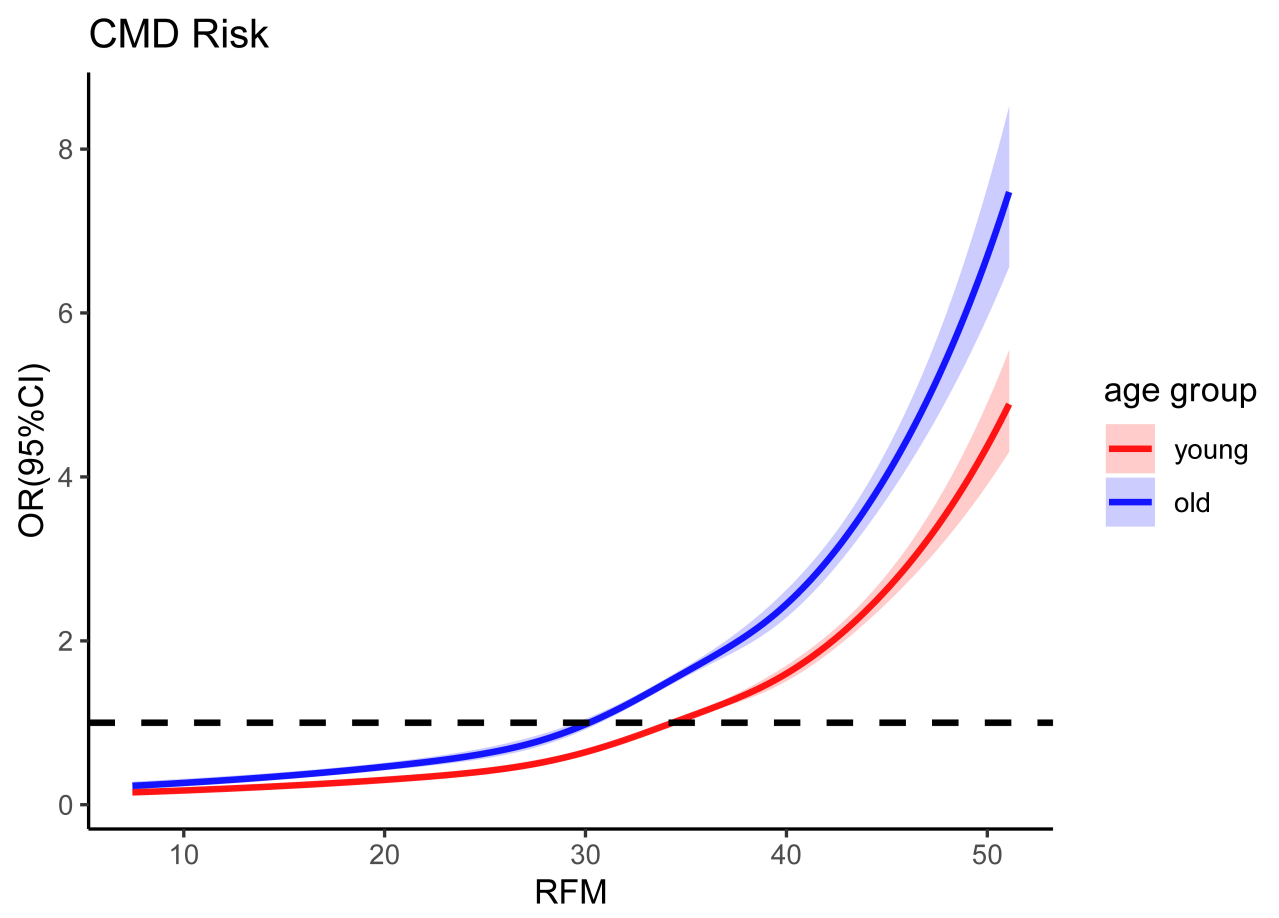
**Dose-response relationship between relative fat mass and cardiometabolic disease risk**. Young: age <60 years group; Old: age ≥60 years group. Abbreviations: CI, confidence interval; CMD, cardiometabolic disease; OR, odds ratio; RFM, relative fat mass. Restricted cubic spline models adjusted for sex, annual household income, educational attainment, alcohol consumption, smoking status, physical activity, diet, hypertension, and dyslipidemia.

Restricted cubic spline analyses adjusted for age, sex, household income, education, smoking, alcohol use, physical activity, diet, hypertension and dyslipidemia revealed distinct patterns across populations (Fig. 3). In the total population, RFM exhibited a J-shaped association with CMD risk (*p* for overall <0.001, *p* for non-linear <0.001). Similar increasing trends were observed in both age groups, with differing curve morphologies. Both the young and middle-aged group (*p* for overall <0.001, *p* for non-linear = 0.030) and older group (*p* for overall <0.001, *p* for non-linear <0.001) displayed J-shaped associations. The older group demonstrated a steeper risk elevation gradient, with CMD risk (OR >1) emerging at RFM >30, whereas the inflection point occurred earlier (RFM ≈ 34) in the young and middle-aged group.

## 4. Discussion

Our study revealed a significant association between RFM and CMD risk, with elevated RFM levels correlating with an increased risk for CMD. More importantly, we identified pronounced age-related disparities in this relationship. Although both young and middle-aged and older adults exhibited non-linear, J-shaped associations between RFM and CMD risk, older adults demonstrated a distinctly elevated vulnerability. Specifically, the older group showed not only a higher overall CMD risk but also a lower inflection point for RFM-associated risk elevation, and steeper increases in CMD risk per unit rise in RFM compared to the young and middle-aged group. These results identify RFM as a clinically relevant biomarker for CMD risk assessment and highlight that the different associations observed in young and middle-aged and older adults, and may inform the development of more stringent RFM control targets and earlier interventional strategies for older adults.

### 4.1 Association Between RFM and CMD Risk

This study is the first to report the association between RFM and CMD risk in Chinese adults, demonstrating a significant positive correlation. Previous studies have linked RFM to risks of coronary heart disease [11], stroke [12], diabetes [13, 14, 15], and metabolic syndrome [16], however, evidence on its association with CMD as a composite outcome remains scarce. CMD includes diseases with shared pathophysiological mechanisms and therapeutic targets, often presenting as comorbidities. For instance, metabolic inflammation serves as a central mechanism linking obesity, insulin resistance, and cardiovascular diseases. Adipose tissue—particularly visceral fat—secretes inflammatory cytokines (e.g., IL-6, TNF-α), activating the TLR4/NF-κB pathway, which induces insulin resistance, endothelial dysfunction, and atherosclerotic plaque formation [27, 28, 29, 30]. This systemic inflammation is not only responsible for the progression of diabetes, but also accelerates coronary heart disease and stroke via oxidative stress and lipid peroxidation. SGLT2 inhibitors, while improving glycemic control, also reduce cardiovascular mortality [31], underscoring potential shared therapeutic targets in CMD. Clinical evidence further supports the numerous co-morbidities associated with CMD: the CAPTURE multinational study found that 32.2% of type 2 diabetes patients had comorbid atherosclerotic cardiovascular disease (ASCVD), including coronary heart disease (16.0%) and cerebrovascular disease (7.7%) [32]. These findings justify analyzing CMD as an integrated entity to optimize comorbidity management and explore common therapeutic strategies. Building on prior research, this study provides critical evidence on the association between RFM and CMD as a composite outcome.

Previous investigations into the associations between RFM and CMD with diabetes, coronary heart disease, and stroke primarily focused on Western populations. This study fills a gap in Chinese evidence while accounting for potential confounders such as physical activity. Zwartkruis *et al*. [11] identified RFM as superior to BMI and waist circumference in predicting the risk of coronary heart disease in a Norwegian cohort of 95,000 adults. Zheng *et al*. [12] reported a positive association between RFM and stroke risk in the U.S. NHANES population, with the highest RFM quartile exhibiting a 44% increased stroke risk (OR = 1.44, 95% CI: 1.09–1.90) compared to the lowest quartile. However, their analysis lacked adjustment for physical activity, a known modifier of cardiometabolic risk [33, 34, 35, 36, 37], potentially influencing outcomes. Cacciatore *et al*. [15] demonstrated RFM’s superior predictive value over BMI for diabetes risk in 1900 older Italian adults. Similarly, Cichosz *et al*. [13] and Suthahar *et al*. [14] found RFM outperformed BMI, waist circumference, and waist-to-hip ratio in predicting diabetes risk in U.S. NHANES and Dutch cohorts, respectively. The present study extends these previous findings by providing robust evidence from a large Chinese population, systematically accounting for physical activity and other potential confounders, thereby offering more generalizable and refined insights into the RFM–CMD relationship.

### 4.2 Age-Specific Differences in the Association Between RFM and CMD

This study identified significant age-related differences in the RFM-CMD risk association between young and middle-aged and older adults, addressing a critical gap in prior research that lacked comparative analyses across age groups. Although Suthahar *et al*. [14] reported stronger associations between RFM and the risk of type 2 diabetes in younger populations (based on higher hazard ratios in the <40-year group), they did not validate the statistical significance of this age-dependent association through formal interaction analyses. Similarly, Zheng *et al*. [12] observed increased RFM-stroke risk correlations in the 20–59-year subgroup (vs. non-significant associations in the 60–85-year group) but provided no mechanistic explanation.

Although the effect sizes for RFM increments were numerically similar between age groups, the significant interaction term, coupled with the different dose-response relationship and inflection points, indicates that the nature of the RFM-CMD association is fundamentally age-dependent. Combined with the higher baseline CMD risk in older adults, even a marginally greater OR per unit increase in RFM can translate into a more substantial increase in absolute risk at higher RFM levels. Therefore, the statistical interaction highlights a critical vulnerability in the elderly: their risk begins to escalate earlier and may compound more rapidly, underscoring the potential value of earlier and more vigilant RFM monitoring in this demographic.

The observed disparities may be partly explained by age-related physiological changes. Aging is associated with ectopic fat deposition in organs such as the liver and muscles, which may exert greater metabolic impact than visceral adipose tissue [18]. Older adults also tend to exhibit elevated baseline inflammatory markers (e.g., IL-6, CRP) [19] and distinct diabetes pathophysiology—primarily driven by β-cell dysfunction in older populations versus insulin resistance in younger groups [20]. These factors, together with RFM’s established correlation with visceral adiposity, may collectively contribute to the divergent RFM–CMD risk patterns across age groups. However, as our study did not include direct biomarker measurements, these mechanisms remain speculative. This study fills a critical evidence gap by demonstrating that while RFM-CMD risk correlations remain positive in both age groups, older adults exhibit steeper risk escalation with RFM elevation and higher absolute CMD risk at elevated RFM levels compared to young and middle-aged individuals.

### 4.3 Dose-Response Relationship Between RFM and CMD Risk

This study provides novel insights into the non-linear dose-response relationship between RFM and CMD risk across multiple age groups, a previously underexplored area. Prior investigations primarily focused on ROC curve analyses comparing RFM’s predictive value against BMI for diabetes [15] and coronary heart disease [13]. While Zheng *et al*. [12] identified non-linear RFM-stroke risk associations using smoothing curve fitting, they did not employ restricted cubic spline analyses for formal dose-response characterization. Through restricted cubic spline modeling, this study revealed significant non-linear associations between RFM and CMD risk in both age groups. These findings advance our understanding of age-specific RFM-CMD risk dynamics, demonstrating distinct inflection points and risk gradients between young and middle-aged and older populations. These results highlight the potential value of establishing age-specific RFM thresholds for risk stratification and suggest that such thresholds may be warranted; however, future validation studies are needed to define clinically applicable cut-offs. This methodology overcomes the limitations of previous approaches by quantifying non-linear relationships while adjusting for confounders, establishing a robust framework for future investigations.

### 4.4 Public Health and Clinical Implications

This study provides scientific evidence for the association between RFM and the risk of CMD, including age-specific patterns, offering a precise, simple, and usable metric for CMD risk assessment while providing age-stratified personalized intervention strategies. RFM, calculated using height, waist circumference, and sex, has demonstrated strong correlations with body fat percentage measured by DXA and BIA [38]. Its cost-effectiveness and ease of implementation compared to DXA/BIA make it a practical tool for evaluation of adiposity. Furthermore, RFM has been shown to outperform traditional anthropometric indices (e.g., BMI, waist circumference) in predicting cardiovascular risk factors [15, 16], cardiometabolic diseases [14, 39], and cardiovascular mortality [40]. The observed age-specific differences in RFM-CMD risk associations underscore the need for age-adapted intervention thresholds, suggesting stricter RFM control targets and intensified CMD risk management for older adults with elevated RFM. These findings provide a scientific foundation for precision prevention and control strategies for CMD.

### 4.5 Limitation

This study has several limitations. First, a key limitation is the reliance on self-reported CMD without independent clinical validation, which may introduce misclassification bias. This potential bias may be more pronounced in older adults, who are more susceptible to under-reporting due to factors such as decreased awareness of asymptomatic conditions or barriers to healthcare access. If present, such non-differential misclassification would likely lead to an underestimation of the true association between RFM and CMD, meaning our observed significant associations are likely conservative estimates of the actual effects. It is important to note that several factors enhance the reliability of our data: the use of trained staff, standardized data collection procedures, and the fact that self-reported medical information was based on prior physician diagnosis. Additionally, the large sample size helps to mitigate the impact of random error. Second, as a cross-sectional study, our design precludes causal inference, and the observed associations should be interpreted as correlations rather than causal effects. Residual confounding may persist despite multivariable adjustments. Third, the absence of inflammatory markers and fat deposition data limits mechanistic exploration. Future research should prioritize incorporating such measures—for instance, using medical imaging to quantify ectopic fat or assays to profile inflammatory cytokines—to validate the proposed hypotheses and elucidate the biological pathways linking adiposity to CMD risk across the lifespan. Future validation studies are needed to define and evaluate age-specific RFM thresholds before they can be considered for clinical implementation. Furthermore, well-designed prospective cohorts, randomized controlled trials, and molecular-level studies are warranted to confirm these associations and elucidate underlying mechanisms and therapeutic targets.

## 5. Conclusions

This large-scale cross-sectional analysis of nearly 100,000 community-dwelling adults in Hunan Province, China, revealed a positive association between RFM and the risk of CMD, characterized by distinct age-specific patterns. Our findings reveal that while both age groups exhibited non-linear, J-shaped dose-response associations between RFM and CMD risk, older adults demonstrated a distinctly elevated vulnerability. These findings enhance the understanding of CMD risk stratification and provide a basis for future research into age-specific RFM thresholds. However, given the cross-sectional design, these results demonstrate association rather than causation. Future prospective studies are needed to establish temporal sequence, validate the potential thresholds, clarify any potential causal relationships, and elucidate underlying biological pathways.

## Data Availability

The datasets used and analyzed during the current study are available from the corresponding author on reasonable request.

## Abbreviations

BIA, Bioelectrical impedance analysis; BMI, Body mass index; ChinaHEART, China Health Evaluation And Risk Reduction Through Nationwide Teamwork; CI, Confidence interval; CMD, Cardiometabolic disease; DXA, Dual-energy X-ray absorptiometry; OR, Odds ratio; RCS, Restricted cubic spline; RFM, Relative fat mass.
